# Evaluation of axial length measurement and other biometric parameters using three different generations of biometers

**DOI:** 10.3389/fmed.2026.1943418

**Published:** 2026-10-07

**Authors:** Ferdinando Cione, Domenico Mucci, Isabella Pellegrino, Francesco Maria Capuano, Maddalena De Bernardo, Nicola Rosa

**Affiliations:** 1 Department of Medicine, Surgery and Dentistry, University of Salerno, Salerno, Italy; 2 Ophthalmological Unit, AOU San Giovanni di Dio e Ruggi D'Aragona, Salerno, Italy

**Keywords:** axial length, biometry, optical low coherence reflectometry, partial coherence interferometry, swept-source optical coherence tomography

## Abstract

**Purpose:**

To compare biometric measurements obtained with partial coherence interferometry (IOLMaster 500, IOLM), optical low-coherence reflectometry (Lenstar 900, LS900), and swept-source optical coherence tomography (Eyestar 900, ES900).

**Methods:**

In this prospective, observational study, axial length (AL), anterior chamber depth (ACD), aqueous depth (AD), lens thickness (LT), and central corneal thickness (CCT) were measured with the three devices. Exclusion criteria were ocular disease, previous ocular surgery, and contact lens use within 72 h. Normality was assessed using the exact Kolmogorov–Smirnov test. Comparisons were performed with paired T-tests and repeated-measures ANOVA with Bonferroni correction. Agreement was evaluated using Bland–Altman plots, and correlation was evaluated using Pearson correlation (*r*), and intraclass correlation coefficients (ICC). Subgroup analysis by eye side was conducted.

**Results:**

Two-hundred-six eyes of 103 healthy subjects (38 females) were recruited at Public hospital “San Giovanni di Dio e Ruggi d’Aragona”, Salerno, Italy. All devices showed excellent correlation for AL and ACD (*r* ≥ 0.947, ICC≥0.939; *p* < 0.001). AL and ACD were comparable between LS900 and IOLM (*Δ*AL = +0.00 ± 0.09 mm; *Δ*ACD = +0.01 ± 0.10 mm; *p* > 0.050). ES900 measured longer AL and deeper ACD than LS900 (*Δ*AL = +0.03 ± 0.10 mm; *Δ*ACD = +0.04 ± 0.11 mm; *p* ≤ 0.031) and IOLM (*Δ*AL = +0.03 ± 0.08 mm; *Δ*ACD = +0.04 ± 0.11 mm; *p* ≤ 0.013). Strong correlation was observed between ES900 and LS900 for CCT, AD, and LT (*r* ≥ 0.979, ICC≥0.961; *p* < 0.001). ES900 measured thicker CCT, deeper AD, and thinner LT (*p* < 0.017).

**Conclusion:**

Despite excellent correlation, ES900 provided systematically higher AL and ACD values and different anterior segment measurements. Therefore, caution should be taken when using these devices interchangeably, especially in longitudinal studies and follow-up evaluations; device-specific interpretation is recommended to optimize refractive outcomes in cataract surgery.

## Introduction

Cataract surgery is one of the most common and successful surgical procedures performed worldwide (1).

Ocular biometry is a noninvasive examination conducted for cataract surgery preparation (2). Biometric parameters are measured before the surgical procedure, allowing the specialist to precisely analyze the length of the eyeball to calculate the optimal IOL for each patient. Additionally, considering the global increase in myopia prevalence, it can be utilized to estimate its progression (3–6).

Accurate preoperative biometric measurements are considered one of the most critical factors for the success of cataract surgeries, especially with the evolution of toric and multifocal intraocular lenses (IOLs) that require the most surgical precision. Ocular biometry can be performed through ultrasound biometry and optical biometry. This examination highlights various specific data suitable for accurately measuring the dimensions of the eyeball, such as:

- Anterior Chamber Depth (ACD), resulting from the union of Central Corneal Thickness (CCT) and Aqueous Depth (AD);
- Lens thickness (LT);
- Total eyeball axial length (AL).

Anterior segment imaging has significantly improved in recent years due to the introduction of new technologies and the evolution of older ones (7–9), including the overcome of group refractive index (GRI) based biometers (10). These improvements enable us to obtain unprecedented qualitative information and quantitative measurements of the cornea, anterior chamber, iridocorneal angle and lens, in addition to an accurate IOL power calculation.

As new or updated biometric devices are introduced into clinical practice, such as swept-source optical coherence tomography (SS-OCT) biometers, it is crucial to thoroughly understand their capabilities, reliability compared to existing devices, and their impact on IOL selection.

SS-OCT is a noninvasive imaging modality that scans biological structures with high resolution, speed, and sensitivity. The output volume of scans allows for three-dimensional analysis of the given structure. The use of SS-OCT technology has proven to be accurate, repeatable, and more consistent in eyes with moderate lens opacity.

Currently, there are devices that integrate tomography and biometry using SS-OCT technology to acquire all the necessary biometric variables for IOL calculation and anterior segment imaging (including corneal tomography), such as the Eyestar 900 (ES900), introduced at the end of 2020.

In clinical practice, when biometric devices fail to produce corneal readings due to highly irregular corneas, corneal topography values are obtained and used to calculate the suggested IOL (11).

The purpose of this study is to compare the ocular parameters measured by the ES900, an SS-OCT based biometer, with those measured by the IOLMaster 500 (IOLM), a validated biometric device based on the principle of partial coherence interferometry (PCI), and Lenstar 900 (LS900), a device based on the principle of optical low coherence reflectometry (OLCR); two of the most commonly used devices for pre-cataract surgical measurements.

The biometric parameters measured by each biometric device were compared to examine their clinical impact.

## Methods

Healthy subjects with no ocular pathologies were recruited for this prospective, observational study during the period from December 2022 to April 2024. The research was carried out in accordance with the principles of the Declaration of Helsinki, and written informed consent was obtained from all participants. Institutional Review Board (IRB) approval was obtained from the reference institution (Cometico Campania, Protocol No. 16544).

Subjects with ocular pathologies such as cataracts, diabetic retinopathy, strabismus, glaucoma, retinal diseases, optic nerve abnormalities, as well as patients who had undergone ocular surgery and pregnant women, were excluded. Additionally, participants were asked not to wear contact lenses within 72 h before the examination. All participants were thoroughly informed about the study's purpose, and written informed consent was obtained from each of them.

Patients underwent an assessment of ocular biometric parameters using three devices:

- IOLM (Carl Zeiss Meditec AG, Jena, Germany, version 5.4.4.00006);
- LS900 (Haag-Streit AG, Koeniz, Switzerland version i9.6.3);
- ES900 (Haag-Streit AG, Koeniz, Switzerland version i9.6.3);

Measurements were obtained by an experienced operator, starting with the right eye (OD) and then the left eye (OS). AL and ACD, measured from the corneal epithelium to the anterior lens surface, were acquired using IOLM, LS900, and ES900. LT, CCT, and AD, measured from the corneal endothelium to the anterior lens surface, were acquired using LS900 and ES900. The order in which the three biometers were used was randomized, and measurements were therefore not systematically obtained using the same device sequence. All measurements were performed in the same room, within the same time period, and under standardized lighting conditions to minimize potential variability related to environmental conditions or time of measurement. For each instrument, the minimum number of measurements required by each device was obtained: five measurements were performed with both the IOLM and LS900, whereas measurements with the ES900 were repeated until satisfactory image quality was achieved according to the device quality criteria.

The collected data were analyzed using Microsoft Excel; statistical analyses were performed using SPSS version 26.0 and R version 4.5.1. Normality of both raw data for each biometric parameter and paired differences evaluated in the comparative analyses was assessed using the Kolmogorov–Smirnov test. Differences between devices were evaluated using repeated-measures ANOVA with Bonferroni *post hoc* correction, or paired T-tests when only two devices were compared. Correlations were assessed using the Pearson correlation coefficient (*r*), and the intraclass correlation coefficient (ICC). Agreement between the different measurement methods was assessed using Bland–Altman analysis, including the calculation of the mean difference and 95% limits of agreement. Statistical significance was defined as *p* < 0.050.

Since bilateral eyes were included, an additional subgroup analysis stratified by eye-side was performed to avoid a type I error and subsequent smaller *p*-values due to inter-eye correlation when both eyes are in the same group (12).

The preliminary required sample size was computed with G*Power software (Version 3.1.9.7) by using the F test family option and considering AL and ACD as the primary endpoints. An *a priori* power analysis was performed using a repeated-measures ANOVA with a within-subject factor, considering one group and three repeated measurements corresponding to the three biometers. Assuming an effect size f of 0.316, based on an explained variance of 0.03 and an error variance of 0.30, and a nonsphericity correction of 1, it was estimated that a sample size of 98 eyes would be necessary, with a significance level of 0.05 and a test power of 0.80.

## Results

Two-hundred-six eyes of 103 subjects (38 females) were recruited, with an average age of 26.88 ± 0.38 years.

Since IOLM does not measure CCT, AD, and LT, the comparison of these parameters was only made between the LS900 and ES900.

Keratometry and other corneal measurements were not further evaluated because it was not the purpose of this study.

All other biometric parameters analyzed in the whole group were reported in Table 1 and in Figures 1–5; descriptive statistics according to eye-side subgroups were reported in Table 2. Table 3 reported the mean bias together with the corresponding 95% limits of agreement, calculated using 1.96 times the standard deviation (SD) of the differences, explicitly for every device comparison for AL, ACD, AD, CCT, LT between instruments.

**Table 1:** Descriptive statistics of analyzed biometric parameters with all devices.

Statistic	ES900		LS900		IOLM	
	AL (mm)	ACD (mm)	AL (mm)	ACD (mm)	AL (mm)	ACD (mm)
Mean ± SD	24.65 ± 1.46	3.76 ± 0.34	24.62 ± 1.47	3.73 ± 0.35	24.62 ± 1.46	3.72 ± 0.33
CI 95%	24.45/24.85	3.72/3.81	24.42/24.82	3.69/3.78	24.42/24.82	3.68/3.77
Median	24.39	3.78	24.35	3.75	24.40	3.74
Variance	2.31	0.12	2.17	0.12	2.13	0.11
Minimum	21.87	2.99	21.85	2.57	21.87	2.78
Maximum	29.30	4.57	29.28	4.55	29.26	4.59
IQR	2.15	0.55	2.20	0.55	2.20	0.54
KS	0.088	0.176	0.089	0.130	0.133	0.517

Statistic	**ES900**			**LS900**		
	**CCT (µm)**	**AD (mm)**	**LT (mm)**	**CCT (µm)**	**AD (mm)**	**LT (mm)**
Mean ± SD	542.33 ± 32.37	3.22 ± 0.35	3.60 ± 0.23	540.55 ± 34.57	3.19 ± 0.34	3.64 ± 0.24
CI 95%	537.88/546.78	3.17/4.01	3.56/3.63	535.80/545.40	3.15/3.24	3.61/3.67
Median	538.50	3.23	3.58	538.00	3.19	3.62
Variance	1,047.94	0.12	0.05	1,195.19	0.12	0.05
Minimum	459.00	2.45	3.10	452.00	2.05	3.11
Maximum	641.00	4.01	4.40	645.00	3.99	4.47
IQR	48.00	0.55	0.29	51.00	0.54	0.27
KS	0.501	0.091	0.367	0.327	0.073	0.212

ES900, Eyestar 900; LS900, Lenstar 900; IOLM, IOLMaster 500; SD, standard deviation; 95% CI, 95% confidence interval around the mean; SD, standard deviation; IQR, interquartile range; KS, normality check through exact Kolmogorov Smirnov Test; AL, axial length; ACD, anterior chamber depth; CCT, central corneal thickness; AD, aqueous depth; LT, lens thickness.

**Figure 1:**
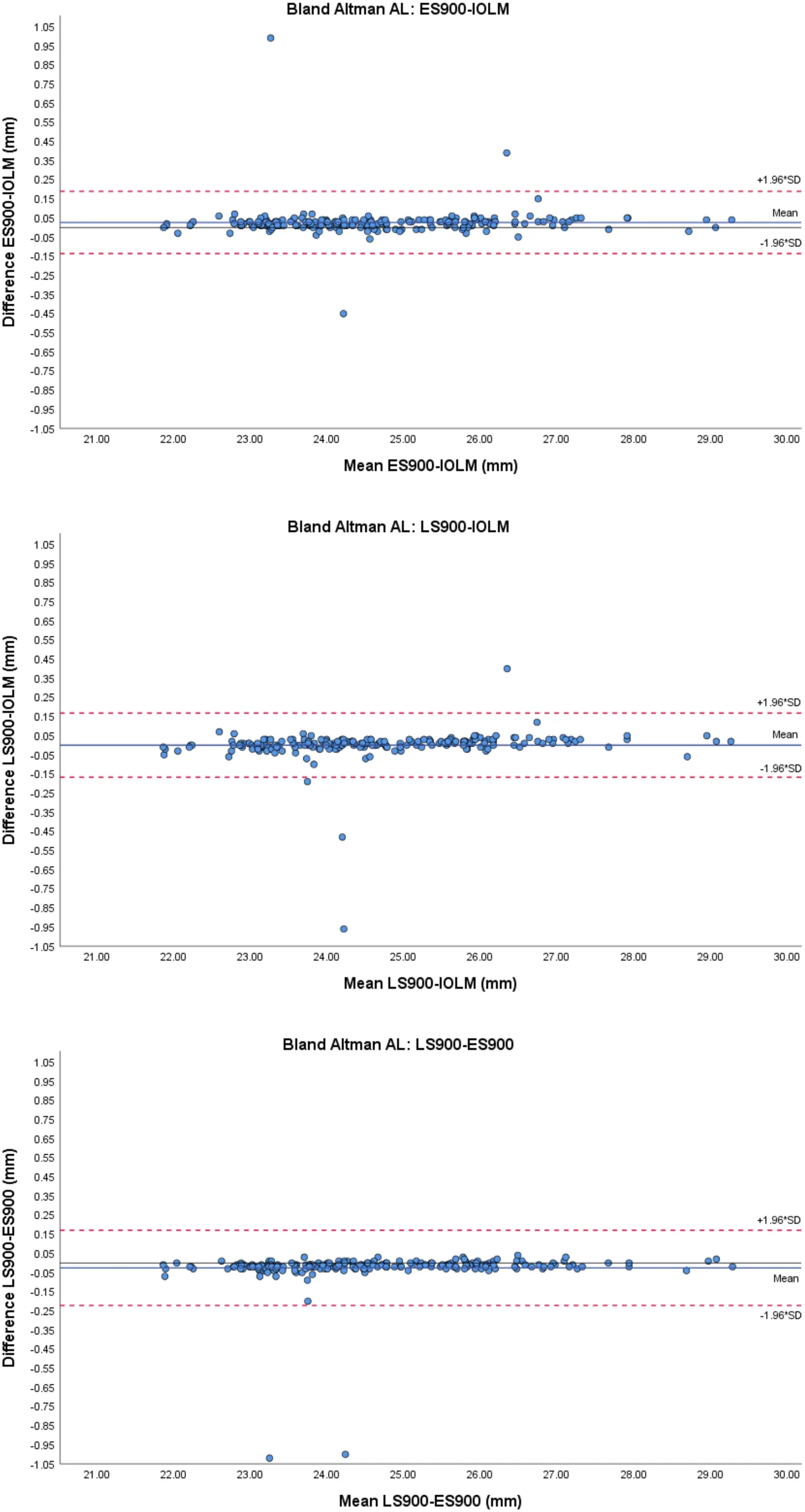
Bland-Altman plot of agreement in axial length (AL) measurements between Eyestar 900 (ES900), Lenstar 900 (LS900), and IOLMaster 500 (IOLM).

**Figure 2:**
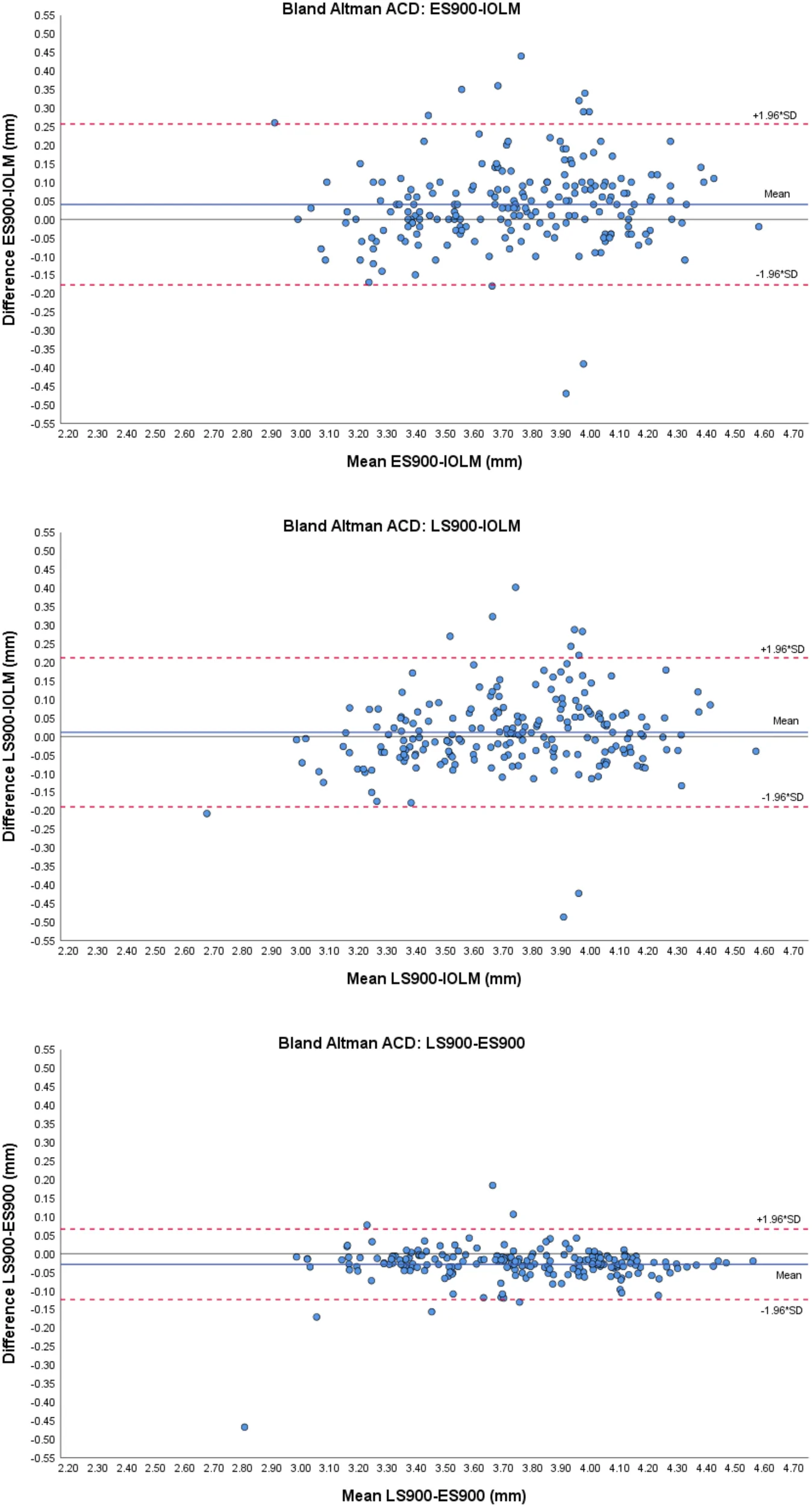
Bland-Altman plot of agreement in anterior chamber depth (ACD) measurements between Eyestar 900 (ES900), Lenstar 900 (LS900), and IOLMaster 500 (IOLM).

**Figure 3:**
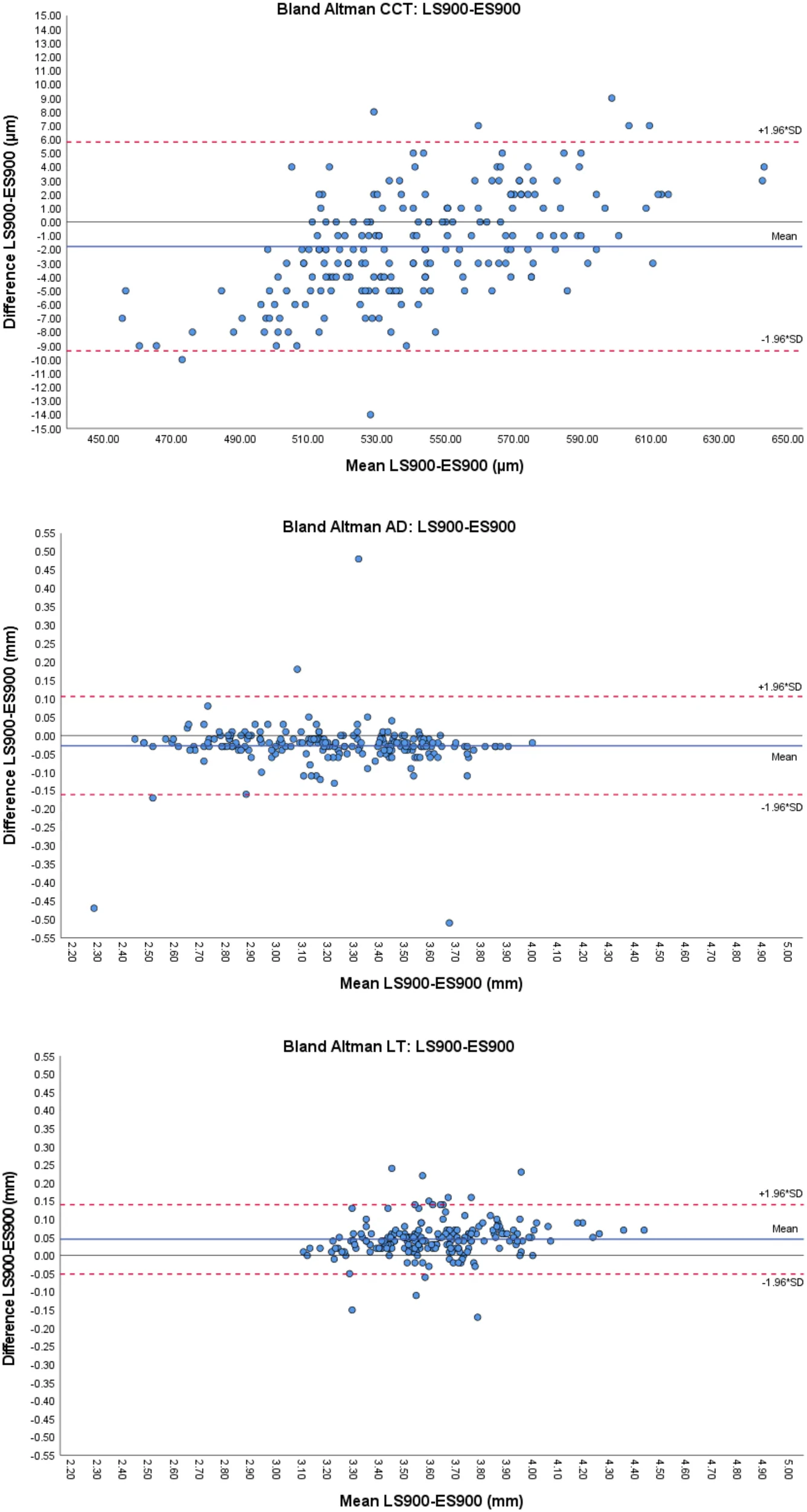
Bland-Altman plot of agreement in central corneal thickness (CCT), aqueous depth (AD) and lens thickness (LT) measurements between Eyestar 900 (ES900) and Lenstar 900 (LS900).

**Figure 4:**
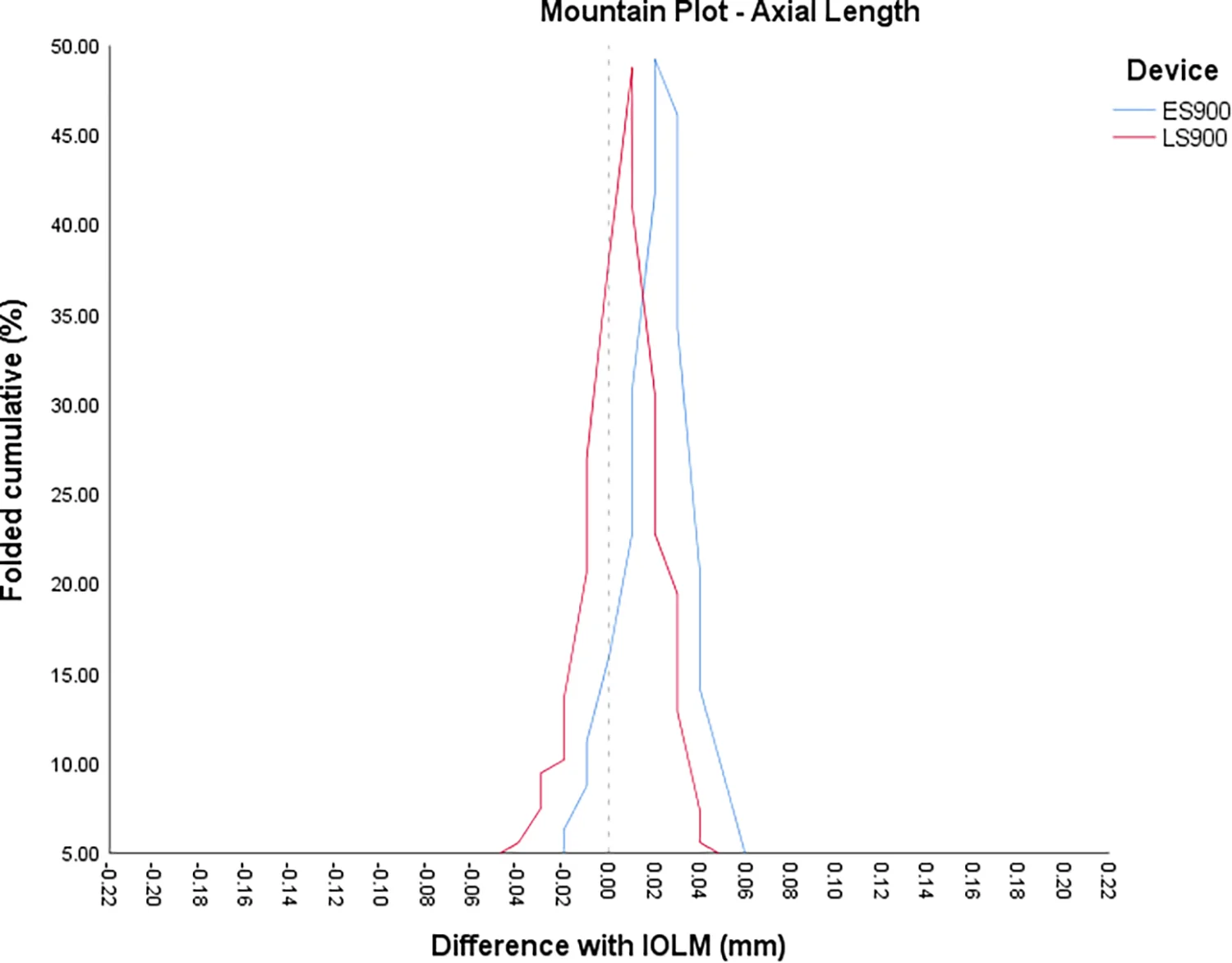
Mountain plot presenting the distribution of the difference in axial length (AL) between Eyestar 900 (ES900), Lenstar 900 (LS900), and IOLMaster 500 (IOLM) measurements. Only folded cumulative percentages ≥5.00% were reported.

**Figure 5:**
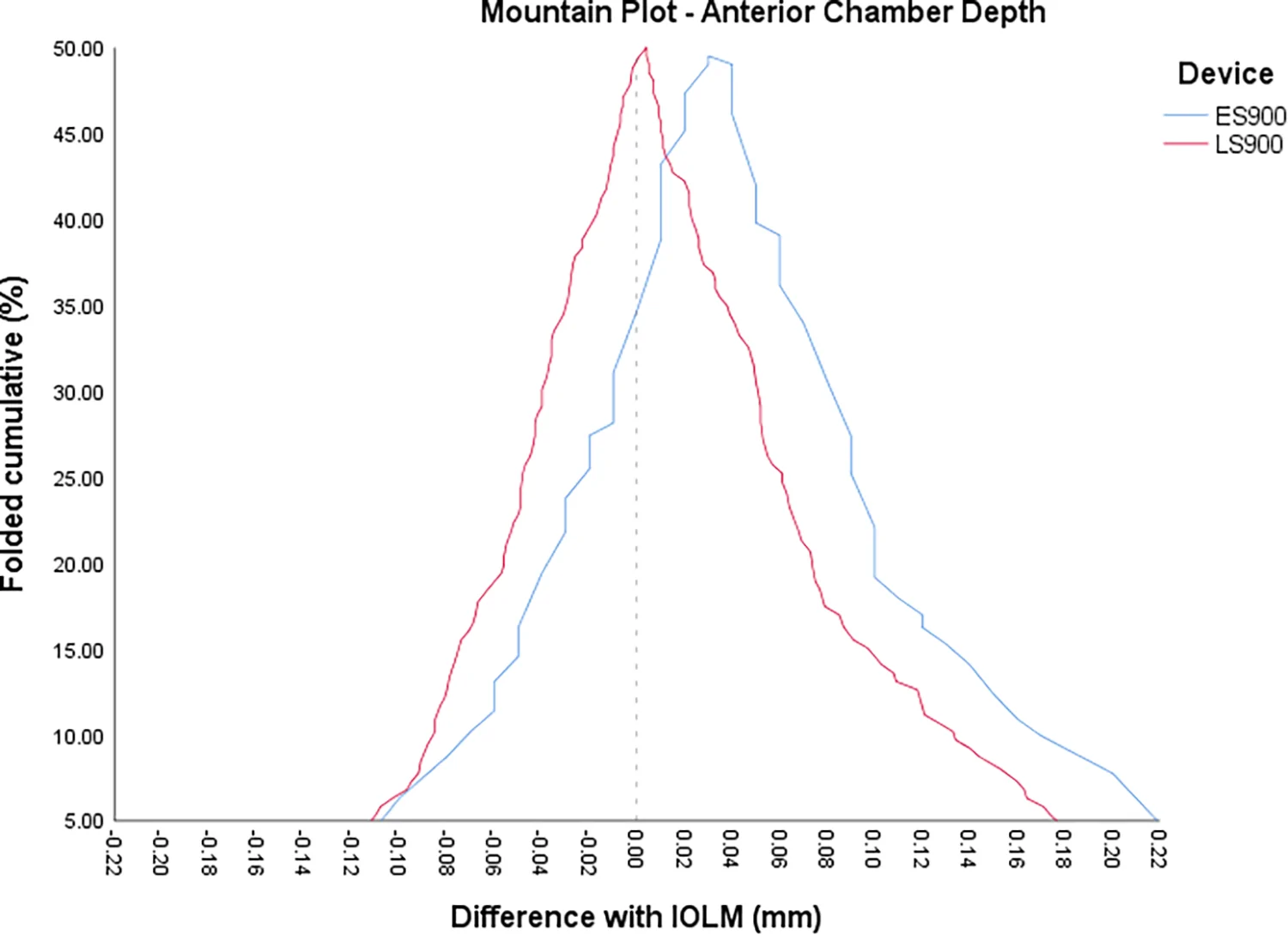
Mountain plot presenting the distribution of the difference in anterior chamber depth (ACD) between Eyestar 900 (ES900), Lenstar 900 (LS900), and IOLMaster 500 (IOLM) measurements. Only folded cumulative percentages ≥5.00% were reported.

**Table 2:** Descriptive statistics of analyzed biometric parameters according to eye side.

OD	ES900		LS900		IOLM	
	AL (mm)	ACD (mm)	AL (mm)	ACD (mm)	AL (mm)	ACD (mm)
Mean ± SD	24.66 ± 1.47	3.76 ± 0.35	24.63 ± 1.48	3.73 ± 0.35	24.63 ± 1.47	3.75 ± 0.34
CI 95%	24.37/24.95	3.69/3.82	24.33/24.92	3.66/3.80	24.34/24.91	3.68/3.81
Median	24.34	3.76	24.23	3.73	24.35	3.75
Variance	2.16	0.12	2.20	0.12	2.15	0.12
Minimum	21.92	2.99	21.85	2.57	21.90	2.78
Maximum	29.30	4.57	29.28	4.55	29.26	4.59
IQR	2.16	0.57	2.23	0.57	2.18	0.60
KS	0.169	0.482	0.155	0.551	0.292	0.700

**OD**	**ES900**			**LS900**		
	**CCT (µm)**	**AD (mm)**	**LT (mm)**	**CCT (µm)**	**AD (mm)**	**LT (mm)**
Mean ± SD	541.62 ± 32.16	3.21 ± 0.35	3.60 ± 0.23	540.68 ± 34.35	3.19 ± 0.35	3.64 ± 0.23
CI 95%	535.34/547.91	3.14/3.28	3.56/3.64	535.97/547.39	3.12/3.26	3.60/3.69
Median	537.00	3.20	3.60	538.00	3.18	3.63
Variance	1,034.081	0.12	0.05	1,179.65	0.12	0.055
Minimum	459.00	2.45	3.12	452.00	2.05	3.14
Maximum	641.00	4.01	4.32	645.00	3.99	4.39
IQR	48.00	0.55	0.29	51.00	0.56	0.27
KS	0.800	0.390	0.527	0.884	0.547	0.458

**OS**	**ES900**		**LS900**		**IOLM**	
	**AL (mm)**	**ACD (mm)**	**AL (mm)**	**ACD (mm)**	**AL (mm)**	**ACD (mm)**
Mean ± SD	24.64 ± 1.46	3.77 ± 0.34	24.62 ± 1.47	3.74 ± 0.34	24.62 ± 1.46	3.70 ± 0.32
CI 95%	24.35/24.92	3.70/3.84	24.34/24.91	3.67/3.80	24.33/24.90	3.63/3.76
Median	24.46	3.80	24.45	3.74	24.44	3.73
Variance	2.13	0.12	2.16	0.11	2.13	0.10
Minimum	21.87	3.03	21.86	2.97	21.87	3.02
Maximum	29.07	4.48	29.09	4.46	29.07	4.37
IQR	2.25	0.55	2.27	0.55	2.23	0.48
KS	0.321	0.517	0.372	0.381	0.429	0.829

**OS**	**ES900**			**LS900**		
	**CCT (µm)**	**AD (mm)**	**LT (mm)**	**CCT (µm)**	**AD (mm)**	**LT (mm)**
Mean ± SD	543.04 ± 32.73	3.23 ± 0.35	3.59 ± 0.23	533.58 ± 34.96	3.20 ± 0.34	3.64 ± 0.25
CI 95%	536.64/549.43	3.16/3.30	3.55/3.64	533.58/547.25	3.13/3.27	3.59/3.69
Median	539.00	3.24	3.57	534.00	3.19	3.61
Variance	1,071.06	0.12	0.05	1,222.42	0.12	0.06
Minimum	459.00	2.49	3.10	454.00	2.43	3.11
Maximum	641.00	3.93	4.40	644.00	3.89	4.47
IQR	48.00	0.55	0.29	54.00	0.54	0.29
KS	0.737	0.447	0.628	0.389	0.240	0.759

OD, right eye; OS, left eye; ES900, Eyestar 900; LS900, Lenstar 900; IOLM, IOLMaster 500; SD, standard deviation; 95% CI, 95% confidence interval around the mean; SD, standard deviation; IQR, interquartile range; KS, normality check through exact Kolmogorov Smirnov Test; AL, axial length; ACD, anterior chamber depth; CCT, central corneal thickness; AD, aqueous depth; LT, lens thickness.

**Table 3:** Bland-Altman mean bias and 95% limits of agreement, for each device comparison.

Parameter	Comparison	Mean bias (CI)	Lower LoA (CI)	Upper LoA (CI)	Outliers (%)
AL (mm)	ES900 vs. IOLM	+0.03 (0.02, 0.04)	−0.14 (−0.16, −0.12)	0.19 (0.17, 0.21)	3 (1.5%)
	LS900 vs. IOLM	+0.00 (−0.01, 0.01)	−0.17 (−0.19, −0.15)	0.17 (0.15, 0.19)	4 (1.9%)
	LS900 vs. ES900	−0.03 (−0.04, −0.01)	−0.22 (−0.25, −0.20)	0.17 (0.15, 0.20)	2 (1.0%)
ACD (mm)	ES900 vs. IOLM	+0.04 (0.03, 0.06)	−0.18 (−0.20, −0.15)	0.26 (0.23, 0.28)	12 (5.8%)
	LS900 vs. IOLM	+0.01 (−0.00, 0.03)	−0.19 (−0.22, −0.17)	0.21 (0.19, 0.24)	10 (4.9%)
	LS900 vs. ES900	−0.03 (−0.04, −0.02)	−0.12 (−0.14, −0.11)	0.07 (0.06, 0.08)	7 (3.4%)
CCT (µm)	LS900 vs. ES900	−1.78 (−2.31, −1.25)	−9.37 (−10.29, −8.45)	5.81 (4.88, 6.73)	7 (3.4%)
AD (mm)	LS900 vs. ES900	−0.03 (−0.04, −0.02)	−0.16 (−0.18, −0.15)	0.11 (0.09, 0.12)	5 (2.4%)
LT (mm)	LS900 vs. ES900	+0.05 (0.04, 0.05)	−0.05 (−0.06, −0.04)	0.14 (0.13, 0.15)	10 (4.9%)

CI, confidence interval; LoA, 95% limits of agreement; outliers (%), numbers and percentage of eyes outside LoA based on 206 paired eyes; ES900, Eyestar 900; LS900, Lenstar 900; IOLM, IOLMaster 500; AL, Axial Length; ACD, Anterior Chamber Depth; CCT, Central Corneal Thickness; AD, Aqueous Depth; LT, Lens Thickness.

There is a high correlation in AL measurement present between ES900 and LS900 (*r* = 0.998, ICC = 0.998; both *p* < 0.001), between ES900 and IOLM (*r* = 0.998, ICC = 0.997; *p* < 0.001) and between LS900 and IOLM (*r* = 0.998, ICC = 0.998; *p* < 0.001). Similar values were obtained with OD and OS subgroups (all *r* ≥ 0.996, all ICC ≥ 0.994; all *p* < 0.001).

There is a high correlation also in the measurement of ACD between ES900 and LS900 (*r* = 0.990, ICC = 0.987; *p* < 0.001), between ES900 and IOLM (*r* = 0.947, ICC = 0.939; *p* < 0.001), and between LS900 and IOLM (*r* = 0.955, ICC = 0.957; *p* < 0.001). Similar values were obtained with OD and OS subgroups (all *r* ≥ 0.943, all ICC ≥ 0.921; all *p* < 0.001) This suggests that AL and ACD measurements obtained from these devices are highly correlated and show close agreement at the population level, although this does not by itself establish interchangeability at the individual-eye level. Repeatability or reliability were not directly assessed in this study.

AL measurements obtained with IOLM and LS900 are superimposable both in the whole group and in the eye-side subgroups (*Δ*AL = +0.00 ± 0.09 mm, *Δ*AL_OD_ = +0.00 ± 0.12 mm, *Δ*AL_OS_ = +0.00 ± 0.03 mm; all *p* > 0.050). ES900 measures longer AL both than IOLM (*Δ*AL = +0.03 ± 0.08 mm, *Δ*AL_OD_ = +0.03 ± 0.11 mm, *Δ*AL_OS_ = +0.02 ± 0.02 mm; all *p* ≤ 0.013), and than LS900 (*Δ*AL = +0.03 ± 0.10 mm, *Δ*AL_OD_ = +0.04 ± 0.14 mm, *Δ*AL_OS_ = +0.02 ± 0.03 mm; all *p* ≤ 0.031).

ACD measurements obtained with LS900 and IOLM are overlapping in the whole database and in OD subgroup, but LS900 measures a deeper value in OS subgroup (*Δ*ACD = +0.01 ± 0.10 mm, *Δ*ACD_OD_ = −0.02 ± 0.09 mm; both *p* > 0.050; *Δ*ACD_OS_ = +0.04 ± 0.11 mm; *p* < 0.001). ES900 measures a deeper ACD compared with IOLM in the whole database and in OD subgroup, but they were comparable in the OS subgroup (*Δ*ACD = +0.04 ± 0.11 mm, *Δ*ACD_OS_ = +0.07 ± 0.11 mm; all *p* ≤ 0.001; *Δ*ACD_OD_ = 0.01 ± 0.10 mm; *p* > 0.050). ES900 measures a deeper ACD compared with LS900 (*Δ*ACD = +0.03 ± 0.05 mm, *Δ*ACD_OD_ = 0.03 ± 0.06 mm, *Δ*ACD_OS_ = +0.03 ± 0.03 mm; all *p* < 0.001).

Figures 4,5 showed graphically the distribution of the difference in AL and ACD between ES900, LS900, IOLM through mountain plot graphs (9).

There is a high correlation in the measurement of AD, (*r* = 0.981, ICC = 0.978; *p* < 0.001), CCT (*r* = 0.995, ICC = 0.991; *p* < 0.001), and LT (*r* = 0.979, ICC = 0.961; *p* < 0.001) between ES900 and LS900, respectively. Similar values were obtained with OD and OS subgroups (all *r* ≥ 0.976, all ICC ≥ 0.961; all *p* < 0.001).

Compared to LS900, ES900 measures a deeper AD (*Δ*AD = +0.03 ± 0.07 mm, *Δ*AD_OD_ = +0.02 ± 0.08 mm, *Δ*AD_OS_ = +0.03 ± 0.06 mm; *p* ≤ 0.003), a thicker CCT (*Δ*CCT = +1.78 ± 3.87 µm, *Δ*CCT_OD_ = +0.94 ± 3.96 µm, *Δ*CCT_OS_ = +2.62 ± 3.61 µm; *p* ≤ 0.017), and a thinner LT (*Δ*LT = −0.04 ± 0.05 mm, *Δ*LT_OD_ = −0.04 ± 0.05 mm, *Δ*LT_OS_ = −0.05 ± 0.05 mm; *p* < 0.001).

## Discussion

The analysis of biometric data was performed using the following three different generations of biometers: PCI (IOLM), OLCR (LS900) and SS-OCT (ES900). IOLM is based on PCI, measuring the transit time of infrared light reflected from the anterior cornea and retinal pigment epithelium along the visual axis (13–16). LS900 uses OLCR technology with a superluminescent diode to measure CCT, AD, ACD, LT and AL, and a separate LED-based system for keratometry and white-to-white distance (17–19). ES900 uses SS-OCT technology, a Fourier-domain interferometric technique that additionally provides full anterior and posterior corneal tomography, pachymetry, and dual-zone reflected keratometry in a single automated acquisition, alongside AL, ACD, AD, LT and WTW.

Currently, to the best of our knowledge, ours is the first study to focus on comparing the measurement of AL among three biometers of three different generations. Several other studies have focused on comparing biometric parameters calculated by different machines, but this is the first to specifically analyze these parameters in healthy patients. Similar studies are present in literature, which have been investigated with different materials and methods (20–32).

Taken together, the available literature on ES900 and other optical biometers reveals two main patterns relevant to our findings. First, statistically significant but small device-dependent biases in AL, ACD, CCT, and LT have been frequently reported across different biometer generations and technologies (20–23, 27, 28). When their impact on IOL power calculation or refractive outcomes has been directly evaluated, these mean differences have generally been considered clinically limited (25, 29, 30). Second, differences observed in individual biometric parameters do not necessarily translate into comparable differences in formula-based IOL power prediction. For example, Multack et al. (29) reported more than 89% of eyes within ±0.50 D across the evaluated devices, while Sorkin et al. (30) found more than 95% of eyes within ±0.50 D. Also Sorkin et al. (32) reported comparable IOL power prediction accuracy between ES900 and IOLMaster 700 using the Barrett Universal II formula. Our findings are consistent with this overall pattern, showing small mean device-dependent differences, with ES900 tending to measure a longer AL and deeper ACD than the other devices. However, the Bland–Altman analysis and the corresponding 95% limits of agreement (Table 3) indicate that individual discrepancies may be larger than suggested by mean differences alone.

To the best of our knowledge, the present study adds several novel contributions. First, by enrolling 206 eyes of 103 healthy patients, it represents one of the largest cohorts directly comparing three different measurement principles:

- PCI: IOLM;
- OLCR: LS900;
- SS-OCT: ES900.

This comprehensive, multi-device design allows not only confirmation of previous trends but also the detection of systematic biases among platforms. Indeed, while all devices showed high correlation for AL and ACD, ES900 consistently yielded slightly higher values for AL and ACD compared to LS900 and IOLM. ES900 also measured thicker CCT, deeper AD, and thinner LT compared to LS900. Therefore, caution should be used when using these devices interchangeably. This concept should be interpreted carefully: in IOL power calculation, the clinical impact of these small differences will probably be limited, despite their statistical significance. To place these differences in clinical context, a formula-based recalculation of IOL power using the raw per-eye dataset was not performed in this analysis. This is a limitation of the study. As an order-of-magnitude estimate, applying the same AL-to-IOL-power conversion ratio reported by Sorkin et al. (25) for the ES900/Anterion comparison (An AL difference of 0.014 mm corresponds to an approximately 0.05 D refractive difference, which translates to about 3.60 D for each mm of AL difference.), the mean AL bias observed here between ES900 and IOLM/LS900 (0.03 mm) would correspond to a theoretical IOL power difference of approximately 0.10 D, below commonly cited clinically insignificant thresholds of 0.25–0.50 D. However, eyes at the upper 95% limit of agreement could show differences approaching 0.70–0.80 D, which would be clinically relevant. This distinction between a small mean-level bias and a clinically relevant range at the individual-eye level supports a more nuanced conclusion than a categorical statement of non-interchangeability. ACD differences may have a greater impact than AL differences. According to recent studies, the IOL power would change by about 0.10 D with 0.10 mm change in ACD (33), but, as reported in Table 3 and in Figure 2, this parameter has a greater fluctuation than AL between biometers: in fact, a greater number of eyes fell outside the upper 95% limit of agreement for ACD than for AL. In addition, it was known that ACD varies across measurement technologies, pharmacological dilation, and ocular surgical procedures (10). As no universally accepted thresholds exist for the other biometric parameters, we avoided assigning arbitrary clinical relevance to the observed differences and instead focused on their magnitude and agreement, without assuming an impact on IOL power or postoperative refractive outcomes.

On the other hand, their relevance may be greater in scientific research, where pooling measurements from different biometers may introduce device-related bias and systematic error, which could also be defined as ‘deterministic’ error (34) and it can conclude the devices cannot be used interchangeably in scientific studies (35, 36). In addition, in longitudinal studies and follow-up, such as the evaluation of myopia progression (37, 38), changing the biometer could mimic or mask a small change in AL.

There are no statistically significant differences in the measurement of AL between LS900 and IOLM. ACD measurements demonstrated to be almost identical between LS900 and IOLM, but statistically significant differences were observed in OS subgroup. Such fluctuations could, in principle, modify effective lens position (ELP) estimation (10) and consequently IOL power calculation, although this was not directly evaluated in the present study. In fact, it has been demonstrated that, in special conditions such as post-refractive surgery eyes (39) and eyes undergoing sutureless scleral fixation with the Yamane technique (40), ACD not only can be omitted from IOL power calculation, but may even be misleading, as inaccurate ACD values can lead to erroneous IOL power estimation (40).

Our study also has limitations: it was conducted exclusively in healthy young subjects, and cataract or other media-opacifying conditions were exclusion criteria: in fact, measurements in young healthy eyes may not fully represent elderly cataract populations (41, 42). Because differences between measurement technologies may be more pronounced in the presence of lens opacity, our findings cannot be directly extrapolated to a cataract-surgery population, and statements regarding optimization of refractive outcomes in cataract surgery should be interpreted as hypothesis-generating rather than as direct clinical evidence. Studies in cataractous eyes are needed before firm interchangeability recommendations can be made for surgical practice.

Another limitation of this study is the inclusion of both eyes from some participants without statistical adjustment for within-subject inter-eye correlation. Although an additional analysis stratified by eye side was performed, this approach does not fully account for the non-independence of bilateral measurements and may therefore have influenced the statistical significance of the observed differences.

One of the strengths is certainly the inclusion of biometers with three different operating methods. On the other hand, all analyzed biometers are GRI-based biometers, therefore a future perspective is given by the comparison of data with a sum-of-segment based biometer (10). In fact, GRI-based biometers convert the measured total optical path length into geometric AL using a single averaged refractive index for the entire eye, whereas sum-of-segments devices measure each ocular segment (cornea, aqueous, lens, and vitreous) separately and apply a medium-specific refractive index to each segment before summing them (10, 43). Because the total optical path is predominantly determined by the vitreous in long eyes, whereas the relative contribution of the anterior segment and lens is greater in short eyes, the use of a single equivalent refractive index may affect AL measurements, particularly in short and long eyes (10). Therefore, the difference between GRI-based and sum-of-segments biometers represents a structural source of measurement discrepancy rather than merely a calibration offset (44).

In conclusion, the analysis of three different generations of biometers showed that although ES900, LS900, and IOLM had excellent correlation for AL and ACD measurements, small but systematic biases were identified between devices. ES900 consistently measured higher AL and ACD values, along with thicker CCT and deeper AD, than the other two devices. Therefore, caution should be taken when using these devices interchangeably. Our findings underscore the importance of device-specific interpretation of biometric data to optimize refractive outcomes in cataract surgery.

## Data Availability

The raw data supporting the conclusions of this article will be made available by the authors, without undue reservation.
